# Mesiodistal angulation of posterior teeth in orthodontic patients with different facial growth patterns

**DOI:** 10.15171/joddd.2019.041

**Published:** 2019

**Authors:** Mohammadreza Badiee, Asghar Ebadifar, Sanaz Sajedi

**Affiliations:** ^1^Dentofacial Deformities Research Center, Research Institute of Dental Sciences, Shahid Beheshti University of Medical Sciences, Tehran, Iran; ^2^Dentofacial Deformities Research Center, Research Institute of Dental Sciences, Shahid Beheshti University of Medical Sciences, Tehran, Iran

**Keywords:** Angulation and posterior teeth, dental occlusion, orthodontics

## Abstract

***Background.*** Mesiodistal and buccolingual angulations of teeth are variable in different malocclusion classes. This study aimed to assess the mesiodistal angulation of posterior teeth in orthodontic patients with vertical, normal, and horizontal facial growth patterns.

***Methods.*** This descriptive, cross-sectional study evaluated 150 lateral cephalograms of orthodontic patients. According to cephalometric analysis, facial growth patterns were divided into three groups of normal, horizontal, and vertical (n=50). The angulation of maxillary and mandibular posterior teeth was then measured. Data were analyzed using SPSS 22.

***Results.*** The results showed an increase in the mean angle of maxillary and mandibular posterior teeth relative to palatal and mandibular planes in patients with a vertical facial growth pattern. Conversely, their angulation decreased relative to the bisected occlusal plane (BOP). The angulation of posterior teeth decreased relative to palatal and mandibular planes and increased relative to the occlusal plane with an increase in overbite. The mean angle of all maxillary teeth relative to the palatal plane was significantly greater in open bite patients compared to normal and deep bite patients. This value in patients with normal bite was significantly greater than that in deep bite patients (P<0.05).

***Conclusion.*** The current results revealed that in patients with a vertical growth pattern, all the posterior teeth had a forward inclination. Conversely, in patients with a horizontal growth pattern, the teeth had a backward inclination.

## Introduction

Malocclusion occurs due to inconsistencies in the complex process of facial growth pattern, environmental factors, and absence of a compensating mechanism in the dentoalveolar arch.^1^ Factors such as age, sex, racial characteristics, facial growth pattern, and some related clinical parameters are essential in orthodontic treatment planning.^2^ Facial growth pattrn is determined at young ages prior to the eruption of maxillary first molars.^3^ Skeletal facial characteristics in individuals with a vertical facial growth pattern include increased overall facial height (especially lower anterior height), clockwise rotation of the mandible, short ramus, and high gonial angle. The skeletal facial characteristics of individuals with horizontal growth pattern are in contrast to those in individuals with vertical growth pattern.^4-7^ Considering the dentoalveolar aspects, the maxillary dental arch in patients with vertical growth pattern is narrower, increasing the chances of posterior crossbite and anterior open bite.^8,9^ Wider dental arch and more severe overbite are seen in patients with a horizontal facial growth pattern.^10^ Tsunori et al^11^ showed that posterior teeth have a buccal inclination in patients with a vertical facial growth pattern. In patients with horizontal growth pattern, posterior teeth have a lingual inclination. Vertical facial growth pattern has some variations. Patients with vertical facial growth pattern are susceptible to deep bite. Patients with a horizontal facial growth pattern might also have anterior open bite.^12-14^

Bjork, in his implant studies, showed that the apical rotation of teeth affects their final position.^15,16^ Kim^17^ reported that in patients with skeletal open bite, posterior teeth have a mesial angulation relative to the occlusal plane, and correction of this mesial angulation is a significant goal in the treatment of patients with open bite. Growth and rotation of the jaws during growth and development affect the path of eruption of the teeth.^18^ A direct association has been noted between the overbite of anterior teeth and their inclination and vertical position of the jaws. It has been reported that the palato-mandibular angle is wider, and open bite is more common in individuals with a long face, and the reverse is true in patients with deep bite.^19^ Bjork^16^ also believed that in patients with open bite, the posterior palate has a downward inclination, causing downward and backward movement of the mandible. Nanda^20^ also confirmed this finding and added that the mandibular, palatal, and anatomic occlusal plane angles decrease during growth and development. This can cause a reduction in the anterior facial height and subsequently less discrepancy in patients with open bite. Ellis and McNamara^21^ reported that increased mandibular angle is associated with open bite and increased lower facial height. They also reported that the occlusal plane is steeper in patients with open bite.^22^ There is a vertical discrepancy (increased anterior facial height) originating from anteroposterior discrepancies (protruded incisors) in most cases of open bite. Thus, anteroposterior discrepancies should also be corrected in order to correct vertical ratios.^23^

According to Enlow et al,^25^ the gonial angle has an anticlockwise rotation to compensate for the posterior movement of the mandible during growth and development, causing a reduction in this angle with aging.^24^ Tsai discussed that incisors are more upright in individuals with a long face. The most stable dentoskeletal pattern in each individual can be achieved when a balance exists between intraoral forces applied by the muscles of mastication, bone, and teeth. In the presence of malocclusion, the sagittal, vertical, and transverse positions of the teeth are in coordination with the base of the skull, creating a stable situation. On the other hand, changing the position of the teeth and jaws in the orthodontic treatment of malocclusions impairs the balance of malocclusion. If a stable situation cannot be achieved between the bone and the surrounding tissues, the treatment results would not be stable.

On the other hand, studies are limited on the relationship between the facial growth pattern and angulation of teeth relative to the alveolar bone and angulation of maxillary and mandibular teeth relative to each other. Previous studies have paid much attention to the dentoalveolar compensation of posterior and anterior teeth in the treatment of open bite, and the mesiodistal angulation of posterior teeth has not been addressed.^26^ Thus, this study aimed to assess the mesiodistal angulation of posterior teeth in patients with different facial growth patterns.

## Methods

This descriptive cross-sectional study evaluated 150 lateral cephalograms retrieved from the archives of the Orthodontic Department of Shahid Beheshti University of Medical Sciences. The study was approved in the Ethics Committee of Shahid Beheshti University of Medical Sciences (IR.SBMU.RIDS.REC.1395.414). The inclusion criteria were high resolution of digital lateral cephalograms, an age range of 14‒20 years, the presence of posterior teeth (the first premolar to the second molar), and permanent dentition period. The exclusion criteria consisted of systemic conditions, the presence of asymmetry on the frontal-view photographs taken at rest, and a history of orthodontic treatment.

A total of 150 lateral cephalograms of patients 14‒20 years of age were retrieved using convenience sampling and divided into three groups according to cephalometric indices.

Group 1 included 50 patients with vertical growth pattern. In this group, the Jarabak index was <62%, the angle between the SN and Me-Go lines was >36°, and the inclination angle was >87°.

Group 2 included 50 patients with a normal vertical growth pattern. The Jarabak index was 62‒65%, the angle between the SN and Me-Go lines was 32‒36°, and the inclination angle was 83‒87°.

Group 3 included 50 patients with a horizontal growth pattern. The Jarabak index was >65%, the angle between the SN and Me-Go lines was <32°, and the inclination angle was <83°.

The angulation of maxillary and mandibular posterior teeth (the vertical axis of the first and second molars and the first and second premolars) with the palatal plane (PNS-ANS), mandibular plane (GO-Me), bisected occlusal plane (BOP), and relative to each other was measured. To assess overbite, first, the magnification of all radiographs was standardized. For this purpose, the magnification of each lateral cephalogram in each group was calculated using a ruler. Next, by taking into account the magnification factor, overbite was measured in all the three groups and standardized. According to the severity of overbite, the samples were divided into three groups of open bite (overbite <1 mm), normal bite (1‒3 mm of overbite), and deep bite (overbite >3 mm).

It should be noted that in premolar teeth, the longitudinal axis of the tooth was considered as the line connecting the apex and cusp tip. In the molar teeth, the longitudinal axis was considered as the line connecting the furcation to the center of the crown.

**Figure 1:**
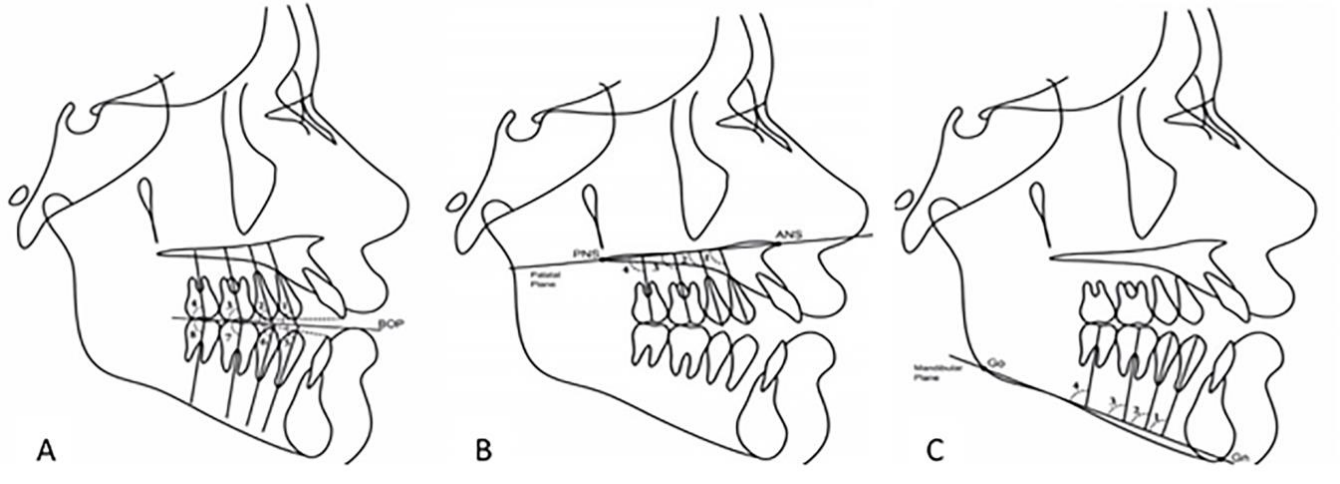
The angle of posterior teeth relative to different planes. A: Maxillary and mandibular teeth to BOP. B: Maxillary teeth to palatal plan. C: Mandibular teeth to mandibular plan.

The BOP was considered as the line connecting the most distant point of molar contact to the midpoint of overbite. Figure 1 shows the angle of posterior teeth relative to different planes.

Data were analyzed using descriptive statistics, including frequency, mean, and standard deviation. Inferential statistics, including one-way ANOVA, were used to compare the angle of posterior teeth in patients with different growth patterns. In the case of the presence of a significant difference, post hoc LSD tests were applied for pairwise comparisons. Pearson’s correlation test was used to assess the correlation of overbite with the angulation of the posterior teeth.

## Results

Table 1 shows the mean parameters measured in the three groups of normal, horizontal, and vertical growth patterns.

**Table 1:** Mean parameters in the three groups of normal, horizontal, and vertical growth patterns (n=50)

**Cephalometric index**	**Mean ± SD**		
	**Normal growth pattern**	**Horizontal growth pattern**	**Vertical growth pattern**
**Jarabak index (%)**	63.50±1.28	70.12±2.53	56.90±2.17
**SN-Me-Go (°)**	33.76±1.02	25.06±1.80	41.72±1.56
**Inclination angle (°)**	84.40±1.35	80.38±2.57	88.48±2.03
**Overbite (mm)**	1.86±1.18	3.96±1.86	1.92±0.02

### Angulation of maxillary posterior teeth relative to the palatal plane

Table 2 shows that the mean angle in patients with vertical growth pattern was significantly higher than that in patients with normal and horizontal growth patterns (P<0.001). Also, in the normal growth pattern, the mean angle was significantly higher than that in the horizontal growth pattern (P<0.001).

**Table 2:** Mean angles of maxillary posterior teeth relative to the palatal plane in the three growth patterns

	**Mean ± SD**			**P-value**
	**Normal growth pattern**	**Horizontal growth pattern**	**Vertical growth pattern**	
**Mx 4. PP (°)**	94.88±1.64	89.04±1.79	97.92±1.17	<0.001
**Mx 5. PP (°)**	89.92±1.93	84.98±1.42	96.20±1.31	<0.001
**Mx 6. PP (°)**	86.48±1.64	81.28±1.78	89.00±1.63	<0.001
**Mx 7. PP (°)**	77.34±1.55	75.30±1.21	80.54±1.77	<0.001

### Angulation of maxillary and mandibular posterior teeth relative to the BOP 

Table 3 shows that the mean angle of all the maxillary and mandibular teeth relative to the BOP in patients with horizontal growth pattern was significantly higher than that in patients with normal and vertical growth patterns (P<0.001). Also, the mean angle of all the maxillary and mandibular teeth in patients with normal growth pattern was significantly higher than that in patients with a vertical growth pattern (P<0.001).

**Table 3:** Mean angles of maxillary and mandibular posterior teeth relative to the BOP

	**Mean ± SD**			**P-value**
	**Normal growth pattern**	**Horizontal growth pattern**	**Vertical growth pattern**		
**Mx 4. BOP (°)**	79.96±1.88	85.48±1.47	74.70±1.62	<0.001
**Mx 5. BOP (°)**	83.04±2.01	89.12±1.24	76.16±1.52	<0.001
**Mx 6. BOP (°)**	88.20±1.96	89.22±1.31	80.30±1.25	<0.001
**Mx 7. BOP (°)**	90.56±1.64	94.42±1.29	82.32±1.43	<0.001
**Md 4. BOP (°)**	83.52±1.83	85.32±1.88	74.74±0.96	<0.001
**Md 5. BOP (°)**	84.82±1.19	88.12±1.24	78.02±1.48	<0.001
**Md 6. BOP (°)**	87.04±1.34	90.70±1.36	79.97±1.38	<0.001
**Md 7. BOP (°)**	88.32±2.07	91.46±1.46	80.72±1.54	<0.001

### Angulation of mandibular posterior teeth relative to the mandibular plane

Table 4 shows that the mean angle of mandibular posterior teeth relative to the mandibular plane in patients with a vertical facial growth pattern was significantly higher than that in patients with normal and horizontal growth patterns (P<0.05). The mean angle of mandibular posterior teeth relative to the mandibular plane in patients with a normal growth pattern was significantly higher than that in patients with a horizontal growth pattern (P<0.001).

**Table 4:** Angles of mandibular posterior teeth relative to the mandibular plane

	**Mean ± SD**			**P-value**
	**Normal growth pattern**	**Horizontal growth pattern**	**Vertical growth pattern**	
**Md 4. MP (°)**	84.16±1.96	80.24±1.19	90.00±1.29	<0.001
**Md 5. MP (°)**	84.10±2.00	79.70±1.45	89.76±1.32	<0.001
**Md 6. MP (°)**	84.14±2.05	79.74±1.38	87.26±1.85	<0.001
**Md 7. MP (°)**	84.10±2.19	79.86±1.56	88.44±2.05	<0.001

As mentioned earlier, to assess the correlation of overbite with angulation of posterior teeth, the patients were divided into three groups with open bite (overbite <1 mm), normal bite (1‒3 mm of overbite) and deep bite (overbite >3 mm). Of patients with a horizontal growth pattern, 38 had deep bite, 11 had normal bite, and one had open bite. Of patients with normal growth pattern, 18 had deep bite, 24 had normal bite, and 8 had open bite. Of patients with vertical growth pattern, 6 had deep bite, 15 had normal bite, and 29 had open bite.

### Correlation of overbite with angulation of maxillary posterior teeth

The amount of overbite had a significant positive correlation with maxillary 4‒7 BOP. An increase in the amount of overbite increased the maxillary 4‒7 BOP (P<0.001). On the other hand, the amount of overbite had a significant but negative correlation with maxillary 4‒7. Increased overbite was correlated with a reduction in maxillary 4‒7 (P<0.001) (Tables 5 and 6).

**Table 5:** Mean angulations of maxillary and mandibular posterior teeth in different groups in terms of overbite

	**Mean ± SD**			**P-value**
	**Normal bite**	**Deep-bite**	**Open-bite**	
**Mx 4. PP (°)**	95.22±1.12	90.12±1.23	97.04±1.54	<0.001
**Mx 5. PP (°)**	89.56±1.27	84.51±1.39	95.55±1.35	<0.001
**Mx 6. PP (°)**	87.14±1.23	80.18±1.47	90.69±1.24	<0.001
**Mx 7. PP (°)**	78.11±1.25	75.10±1.21	81.55±1.28	<0.001
**Md 4. MP (°)**	86.01±1.58	81.04±1.65	91.00±1.29	<0.001
**Md 5. MP (°)**	85.10±1.03	80.70±1.22	89.24±1.78	<0.001
**Md 6. MP (°)**	85.14±1.85	78.74±1.86	87.54±1.89	<0.001
**Md 7. MP (°)**	85.10±1.19	78.57±1.78	87.31±2.15	<0.001

**Table 6:** Angulations of maxillary and mandibular posterior teeth relative to the BOP in open-bite, normal bite and deep-bite patients

	**Mean ± SD**			**P-value**
	**Normal bite**	**Deep-bite**	**Open-bite**	
**Mx 4. BOP (°)**	80.96±1.51	86.21±1.22	75.10±1.52	<0.001
**Mx 5. BOP (°)**	83.54±1.19	90.01±1.12	75.06±1.24	<0.001
**Mx 6. BOP (°)**	89.22±1.85	90.22±1.58	80.25±1.60	<0.001
**Mx 7. BOP (°)**	90.96±2.14	94.96±1.25	83.12±1.17	<0.001
**Md 4. BOP (°)**	83.12±1.58	86.02±2.01	75.26±1.96	<0.001
**Md 5. BOP (°)**	85.74±1.22	88.56±2.03	78.28±1.83	<0.001
**Md 6. BOP (°)**	88.14±1.85	91.10±1.40	80.10±1.14	<0.001
**Md 7. BOP (°)**	88.12±1.17	91.58±1.12	80.72±1.54	<0.001

### Correlation of overbite with angulation of mandibular posterior teeth

The amount of overbite had a significant and positive correlation with mandibular 4‒7 BOP. An increase in the amount of overbite increased the mandibular 4‒7 BOP (P<0.001). The amount of overbite had a significant but negative correlation with mandibular 4‒7 MP. Increased overbite was correlated with a reduction in mandibular 4‒7 MP (P<0.001) (Tables 5 and 6).

## Discussion

This study assessed the mesiodistal angulation of posterior teeth in patients with vertical, normal, and horizontal facial growth patterns. The results showed the highest frequency of deep bite in patients with a horizontal growth pattern (61.2%), while the highest frequency of open bite was noted in patients with a vertical growth pattern (76.3%), which was consistent with the findings of a previous study.^10^ Some other studies have reported that patients with a vertical facial growth pattern are prone to posterior crossbite and anterior open bite.^8,9^ Overbite is seen with greater severity in a horizontal facial growth pattern.^10^

Evidence shows that the angulation of teeth in mesiodistal and buccolingual directions is different in different malocclusion classes.^26^ The current results showed that the mean angulation of maxillary and mandibular posterior teeth, relative to the palatal and mandibular planes in different individuals, changes, so that with an increase in the facial height, the magnitude of these angles increases, and conversely, these angulations decrease relative to the BOP with an increase in the facial height. Moreover, the results of the assessment of the correlation of overbite and angulations of teeth revealed that the angle of posterior teeth relative to the palatal and mandibular planes decreased with an increase in overbite, while their angulation increased relative to the occlusal plane. On the other hand, the current study showed that the mean angle of all the maxillary teeth, relative to the palatal plane in open bite patients, was significantly greater than that in patients with normal bite and deep bite. Moreover, the values in patients with a normal bite were significantly greater than those in patients with deep bite. Some studies, similar to the current investigation, reported variable tooth angulations for different malocclusion classes. For instance, Tsunori et al^11^ showed that in patients with a vertical facial growth pattern, the posterior teeth had a buccal inclination while the posterior teeth had a lingual inclination in subjects with a horizontal growth pattern of the face.^11^ Janson et al^27^ compared the angulation of posterior teeth in patients with open bite and normal occlusion and showed that in open bite patients, the maxillary and mandibular premolars had a mesial angulation relative to the occlusal plane. In contrast, the first and second molars had a distal angulation relative to the mandibular and palatal planes. Maxillary and mandibular premolars had a greater mesial angulation relative to the BOP. This finding was in agreement with our results to some extent. Another study assessed the buccolingual inclination of posterior teeth in subjects with horizontal and vertical growth patterns of the face and reported results similar to ours; however, some of their findings were not statistically significant.^28^

It means that uprighting the teeth in order to close the bite in open bite patients and their forward inclination in deep bite patients can be performed to achieve this goal. The difference between their study and ours was that we evaluated different facial growth patterns. Also, we measured the angulations of teeth in patients with open bite, normal bite, and deep bite. In another cephalometric study, Janson et al^29^ evaluated changes in the angulation of posterior teeth in permanent dentition patients with open bite under extraction and non-extraction orthodontic treatment with vertical elastics in the anterior region. They demonstrated that mandibular posterior teeth had become upright in both groups with both treatment protocols. Thus, the correction of open bite with extraction and non-extraction orthodontic treatment protocols with archwires and vertical and anterior elastics resulted in the uprighting of mandibular posterior teeth. They compared post-treatment changes with the baseline state, while in our study, the angulation of posterior teeth in their natural state was evaluated to assess the dental compensations that might have occurred in this state.

As mentioned earlier, the palatal and mandibular planes are different in different growth patterns. For instance, in an open bite patient, the palatal and mandibular planes are steep, and consequently, the teeth have a forward inclination. In other words, the teeth have a mesial inclination relative to the palatal plane in an open bite patient, and the palatal and mandibular planes are steeper. The current results also confirmed this statement since the teeth had an inclination in open bite patients and were upright in deep bite patients. However, some controversial results were also noted in some groups. For instance, one open bite patient did not show any dental changes. In other words, the dentoalveolar system had compensated the severe steepness of the planes. The reverse was also true in some cases. For instance, one patient with a horizontal facial growth pattern did not have dental deep bite because the teeth had a slight forward inclination. This was also noted in patients with a normal growth pattern. For instance, one patient with a normal growth pattern had open bite. In this patient, the teeth had severe inclination. Another patient with a normal growth pattern had deep bite. In this patient, the teeth had milder inclination and were more upright. The reason why dentoalveolar compensation did not occur in all the individuals was probably the selected age group of patients, which was 12‒20 years. Thus, we did not witness much dentoalveolar compensation. However, long-term follow-up of these patients until older ages might have revealed different results concerning angulation and inclination of the teeth.

Finally, it might be concluded that clinically, in patients with a vertical growth pattern and no anterior open bite, dental compensations occur to upright the teeth. Conversely, in patients with a horizontal growth pattern and no anterior deep bite, teeth have inclinations. This is also true in patients with a normal growth pattern and open bite or deep bite. Thus, uprighting of the teeth can be performed to close the bite in open bite patients, or the teeth might be inclined forward in deep bite patients. Similar studies on a larger sample size with older age are required to assess the role of dental compensations in older age groups. Also, simultaneous assessment of mesiodistal and buccolingual angulations of the teeth is recommended in patients with symmetrical and asymmetrical face and those with posterior crossbite.

## Conclusion

The current results revealed that in patients with a vertical growth pattern, all the posterior teeth had a forward inclination. Conversely, in patients with a horizontal growth pattern, the teeth had a backward inclination. Clinicians can use these parameters in treatment planning and mechanotherapy.

## Acknowledgements

The authors would like to thank the Dentofacial Deformities Research Center, Research Institute of Dental Sciences, Shahid Beheshti University of Medical Sciences for their support.

## Competing Interests

The authors declare no conflict(s) of interest related to the publication of this work.

## Authors’ Contributions

MB: Study conception and design; SS: Acquisition of data, AE: Analysis and interpretation of data, drafting of manuscript and critical revision. All authors have read and approved the final manuscript.

## Funding

Dentofacial Deformities Research Center, Research Institute of Dental Sciences, Shahid Beheshti University of Medical Sciences.

## Ethics Approval

The study was approved in the Ethics Committee of Shahid Beheshti University of Medical Sciences (IR.SBMU.RIDS.REC.1395.414).
